# Calmodulin 2 Facilitates Angiogenesis and Metastasis of Gastric Cancer *via* STAT3/HIF-1A/VEGF-A Mediated Macrophage Polarization

**DOI:** 10.3389/fonc.2021.727306

**Published:** 2021-09-15

**Authors:** Ganggang Mu, Yijie Zhu, Zehua Dong, Lang Shi, Yunchao Deng, Hongyan Li

**Affiliations:** ^1^Department of Gastroenterology, Renmin Hospital of Wuhan University, Wuhan, China; ^2^Key Laboratory of Hubei Province for Digestive System Disease, Renmin Hospital of Wuhan University, Wuhan, China; ^3^Department of Nephrology, Renmin Hospital of Wuhan University, Wuhan, China

**Keywords:** gastric cancer, calmodulin 2, tumor-associated macrophages, angiogenesis, cancer

## Abstract

**Background:**

Tumor-associated macrophages (TAMs) are indispensable to mediating the connections between cells in the tumor microenvironment. In this study, we intended to research the function and mechanism of Calmodulin2 (CALM2) in gastric cancer (GC)-TAM microenvironment.

**Materials and methods:**

CALM2 expression in GC tissues and GC cells was determined through quantitative real-time PCR (qRT-PCR) and immunohistochemistry (IHC). The correlation between CALM2 level and the survival rate of GC patients was assessed. The CALM2 overexpression or knockdown model was constructed to evaluate its role in GC cell proliferation, migration, and invasion. THP1 cells or HUVECs were co-cultured with the conditioned medium of GC cells. Tubule formation experiment was done to examine the angiogenesis of endothelial cells. The proliferation, migration, and polarization of THP1 cells were measured. A xenograft model was set up in BALB/c male nude mice to study CALM2x’s effects on tumor growth and lung metastasis *in vivo.* Western Blot (WB) checked the profile of JAK2/STAT3/HIF-1/VEGFA in GC tissues and cells.

**Results:**

In GC tissues and cell lines, CALM2 expression was elevated and positively relevant to the poor prognosis of GC patients. In *in-vitro* experiments, CALM2 overexpression or knockdown could facilitate or curb the proliferation, migration, invasion, and angiogenesis of HUVECs and M2 polarization of THP1 cells. In *in-vivo* experiments, CALM2 boosted tumor growth and lung metastasis. Mechanically, CALM2 could arouse the JAK2/STAT3/HIF-1/VEGFA signaling. It was also discovered that JAK2 and HIF-1A inhibition could attenuate the promoting effects of CALM2 on GC, HUVECs cells, and macrophages.

**Conclusion:**

CALM2 modulates the JAK2/STAT3/HIF-1/VEGFA axis and bolsters macrophage polarization, thus facilitating GC metastasis and angiogenesis.

## Introduction

Gastric cancer (GC) is the second leading cause of cancer-concerned mortality and the fourth most prevalent cancer across the world (1). Tumor microenvironment (TME) refers to the internal environment where tumor cells form and live, covering not only tumor cells but also fibroblasts, immune and inflammatory cells, glial cells, and other cells. By releasing all kinds of molecules, TME in GC can facilitate GC cells’ angiogenesis, invasion, metastasis, and chronic inflammation (2, 3). Recent studies have disclosed that tumor-correlated macrophages (TAM) are the most common immune cells in TME. It is believed that these cells, polarized into M2-phenotype, enhance tumor development and pertain to poor tumor prognosis (4, 5). Inhibited M2-phenotype macrophage polarization can hinder the malignant progression of cancers (6–8). Therefore, it’s very important to understand the underlying mechanism of TAM exerting its carcinogenic function in GC.

Calmodulin (CALM) is a Ca^2+^ binding protein consisting in all eukaryotic cells. After being bonded to target proteins, it can modulate the activities of numerous enzymes, pathways, signal transduction, adaptors, and structural proteins and regulate the functions of signal transduction pathways, hence controlling tumor cells’ migration and invasion (9). CALM is encoded by a multigene family consisting of three members: CALM1, CALM2, and CALM3. CALM2, 1377 bp in length, is situated at 2p21.1-p21.3. CALM2 mutation is connected with arrhythmia (10, 11). CALM2, to a large extent, correlates with anaplastic large cell lymphoma (12), breast cancer (13), and other diseases. Park Sy et al. have denoted that CALM2 inhibition can hamper cell proliferation and colony formation in hepatocellular carcinoma (HCC) cells by inducing apoptosis (14). Given the above findings, CALM2 is associated with cancer progression, but the correlation between CALM2 and GC remains to be seen.

Signal transducer and activator of transcription 3 (STAT3), a cytoplasmic transcription factor, is able to modulate cell proliferation, differentiation, apoptosis, angiogenesis, inflammation, and immune responses (15). Janus kinases (JAKs) are critical to cytokine receptor signaling transduction. The JAK2/STAT3 pathway activated in GC can cramp malignant GC progression when it’s suppressed (16). How the cells react to oxygen levels in solid tumors is monitored by hypoxia inducible factor-1 (HIF-1). In GC patients, long-term hypoxia contributes to HIF-1A activation, which is intricately related to aggressive tumor phenotypes and poor prognosis (17).

Vascular endothelial growth factor (VEGF), a homodimer glycoprotein, is a pivotal mediator of angiogenesis in cancer (18). VEGFA, a member of the VEGF family, presents a high expression in GC tissues and cell lines. VEGFA inhibition can dampen GC cells’ growth, migration, and invasion (19). Hyperthermia can repress the EGFR/STAT3/HIF-1A/VEGF-A axis, thereby abating glioma cell proliferation (20). There have been some reports on the profiles and functions of STAT3, HIF-1A, and VEGF-A in GV. Notwithstanding, we are still in the dark about how the STAT3/HIF-1A/VEGF-A signaling pathway functions in GC.

Here, we tried to have an insight into the function of CALM2 in GC. It was uncovered that CALM2 was prominently up-regulated in gastric cancer tissues, and CALM2 overexpression considerably enhanced GC cell proliferation and metastasis. Therefore, we have furthered our study to investigate whether CALM2 can display its function in GC by restraining STAT3/HIF-1A/VEGF-A, which may provide a new approach for GC treatment.

## Materials and Methods

### Collection of Clinical Samples

From June 2017 to December 2017, 31 samples of gastric cancer tissues and their paired adjacent normal tissues were gleaned from the patients who had received no preoperative treatment before in the People’s Hospital of Wuhan University. They signed the informed consent with a full understanding of how the specimens would be used. The study, authorized by the ethics committee of People’s Hospital of Wuhan University, was implemented in accordance with the Helsinki Declaration and Code of Clinical Practice.

### Immunohistochemistry

GC tumor tissues and xenograft tumor tissues were routinely embedded in paraffin and severed into slices (4 μm). After being dewaxed with xylene and hydrated with gradient alcohol, they were blocked with 3% H_2_O_2_ for 10 minutes to get the endogenous peroxidase inactivated. For microwave reparation (pH=6.0, 15 minutes), 0.01 mol/L sodium citrate buffer solution was administered. Following 20 minutes’ blocking with 5% bovine serum albumin (BSA), the samples were incubated overnight along with primary antibodies Anti-CALM2 (1:100), Anti-CD163 (1:100), Anti-CD206 (1:100), Anti-CD11b (1:100), Anti-CD31 (1:100), Anti-VEGFA (1:100), and Anti-ki67 (1:100) at 4°C, which were all supplied by Abcam. The next day, the secondary antibody Goat anti-Rabbit IgG was added for 20 minutes’ incubation at indoor temperature. DAB (DAB Beijing Zhongshan Jinqiao Biological Company) was taken for coloring subsequent to PBS washing. The samples were redyed with hematoxylin, dehydrated, made transparent, and sealed for microscopic examination. Three slices were taken from each tumor tissue for detection. With five independent fields chosen randomly, the Olympus microscope (×400) was deployed to count the number of positive cells. The nucleus, cytoplasm, or membrane of the selected positive cells must be tinged with pale brown. The IHC-positive cells in the 400-fold field were counted, and the average was calculated.

### Cell Culture

Human umbilical vein endothelial cells (HUVECs), human mononuclear cells (THP1), and the cells of the human gastric epithelial cell line GES-1 and gastric cancer cell lines SGC7901, BGC823, MKN45, HGC27, and AGS, were acquired from the Cell Center of the Chinese Academy of Sciences (Shanghai, China). The cells were grown with the RPMI1640 (Thermo Fisher Scientific, MA, USA) culture solution incorporating 10% fetal bovine serum (FBS) (Thermo Fisher Scientific, MA, USA) and 1% penicillin/streptomycin (Invitrogen, CA, USA) at 37°C in an incubator with a volume fraction of 5% CO_2_. For cell digestion and passage, 0.25% trypsin (Thermo Fisher HyClone, Utah, USA) was administered in the logarithmic growth phase.

### Cell Transfection

Lipofectamine^®^ 3000 (Invitrogen; Thermo Fisher Scientific, Inc.) was adopted to transfect CALM2-siRNA and its control (si-NC) or CALM2 overexpression plasmid and its control vector (NC-vector) into BGC823 and MKN45 cells, as instructed by the supplier. Twenty-four hours later, the transfection efficiency was ascertained through qRT-PCR and Western Blot.

### Collection of Conditioned Medium (CM)

As previously mentioned (21), AGS and MKN45 cells transfected with CALM2 overexpression plasmid and si-CALM2 reached 80% confluence, after which they were flushed in PBS twice and cultured with a serum-free medium overnight. The supernatant was collected with the cell fragments removed through five minutes’ centrifugation at 16,000 xg. It was stored at -80°C for subsequent experiments.

### Real-Time Quantitative Polymerase Chain Reaction (qRT-PCR)

AGS and MKN45 cells following 24 hours’ transfection were gathered. 1 m TRIzol (Invitrogen, Carlsbad, CA, USA) was added to every 5×10^6^ cells to lyse the cells and extract the total RNA. 2 µg of RNA was taken and transcribed into cDNA in line with the instructions of the RNA reverse transcription kit, and PCR amplification was conducted with cDNA as the template. The following primers were exploited: *CALM2*: forward primer 5’-CTTCAGTCAGTTGGTCAGCC-3’, reverse primer 5’-GAGGTGTTTATGAGGCGCTG-3’; *CXCL12*: forward primer 5’-GGGAACAGTGCATGCATCAA-3’, reverse primer 5’-GGACTCTCAGGACCAAAGCT-3’; *IL-4*: forward primer 5’-GTGTTCTTGGAGGCAGCAAA-3’, reverse primer 5’-GCCTCACATTGTCACTGCAA-3’; *IL-13*: forward primer 5’-GATGCTCCATACCATGCTGC-3’, reverse primer 5’-GGATAAGGGGCGTTGACTCA-3’; *IL-10*: forward primer 5’-ATAGAGTCGCCACCCTGATG-3’, reverse primer 5’-GGCGCTGTCATCGATTTCTT-3’; *VEGFA*: forward primer 5’-CTCACACACACACCAACCAGG-3’, reverse primer 5’-GAAGAAGCAGCCCATGACAG-3’; Internal parameter GAPDH: forward primer 5’-CGCTGAGTACGTCGTGGAGTC-3’, reverse primer 5’-GCTGATGATCTTGAGGCTGTTGTC-3’. qPCR was done as the following: 5 minutes’ pre-denaturation at 95°C; 15 seconds’ denaturation at 95°C, a minute’s annealing at 60°C, 40 cycles in total; 30 seconds’ extension at 95°C and then 15 seconds’ extension at 60°C. The relative expressions of the genes to be examined were presented as the 2^(-ΔΔCt)^ value. The experiment was repeated three times.

### Western Blot (WB)

Total protein was extracted out of cells and animal tissues, as stipulated by the manufacturer. The BCA method was adopted to determine the protein concentration, and the sample was stored for use at -80°C. After denaturation, 20 μg of the total protein was given to each well. The protein was separated through 10% SDS-PAGE gel electrophoresis and then moved onto a PVDF membrane at a constant current of 300 mA. TBST solution incorporating 5% skimmed milk was employed to block the membrane for one hour at room temperature. A blocking solution was utilized to dilute the proteins of the following primary antibodies: Antibody E-cadherin (Abcam, ab40772, 1:1000, MA, USA), Antibody N-cadherin (Abcam, ab76011, 1:1000), Antibody Snail (Abcam, ab216347, 1:1000), Antibody JAK2 (Abcam, ab108596, 1:1000), Antibody p-JAK2 (Abcam, ab195055, 1:1000), Antibody STAT3 (Abcam, ab68153, 1:1000), Antibody p-STAT3 (Abcam, ab267373, 1:1000), Antibody HIF-1 (Abcam, ab51608, 1:1000), Antibody VEGFA (Abcam, ab52917, 1:1000), Antibody CALM2 (Sigma-Aldrich, WH0000805M1, 1:500), Antibody CD206 (Abcam, ab64693, 1:1000), Antibody CD11b (Abcam, ab133357, 1:1000), Antibody CD163 (Abcam, ab182422, 1:1000), Antibody CD86 (Abcam, ab239075, 1:1000), Antibody CD80 (Abcam, ab134120, 1:1000), Antibody iNOS (Abcam, ab178945, 1:1000), and Antibody GAPDH (Abcam, ab9485, 1:1000), which were then incubated with the sealed membrane overnight at 4°C. After being rinsed in TBST four times, 8 minutes each, the membrane was incubated with corresponding secondary antibodies for one hour and a half at indoor temperature (diluted concentration: 1:2000) and then again washed in TBST four times, 8 minutes each. The Thermo’s Pierce ECL Western Blot Substrate kit was deployed for X-ray development.

### CCK8 Experiment

As stipulated by the manufacturer, AGS and MKN45 cells post transfection and macrophages cultivated with the conditioned medium were inoculated into a 96-well plate with a density of 2×10^3^ cells/well and cultured at 37°C with 100% humidity and 5% CO_2_ for 48 hours. 10 μL of CCK8 (Dojindo Molecular Technologies, Kumamoto, Japan) was administered to each hole for one-hour incubation at 37°C, with the absorbance gauged at 450 nm. Each experiment was repeated three times, and each measurement was conducted three times. Then the absorbance of the cells was examined on the 24^th^, 48^th^, and 72^nd^ hours.

### Colony Formation Assay

GC cells (AGS and MKN45) seeded into a 6-well plate with a density of 500 cells/well were cultured for 10 days to form cell colonies. The cells were rinsed with PBS twice and fixed with 4% paraformaldehyde for 15 minutes. Crystal violet was applied to dye the fixed cell colonies for 10 minutes, which were later photographed and counted.

### Transwell Assay

Transwell was implemented as per the method adopted by Wang R (22) et al. In a nutshell, 2×10^4^ transfected AGS and MKN45 GC cells and macrophages grown with the conditioned medium were placed in the upper Transwell compartment (Corning, NY, USA), while 600 μl of the culture solution encompassing 20% FBS was poured into the lower chamber. The cells were cultured at 37°C. Twelve hours later, the cells in the upper room were cleared. The lower chamber cells were fixed with 4% paraformaldehyde and stained with 0.1% crystal violet. After drying, they were photographed and counted. Cell invasion assay: the upper room was coated with Matrigel (8 µm pore size; Corning, Beijing, China) before it was removed, and then cells could be added; the other procedures were the same as done in the migration assay.

### Tubule Formation Experiment

As mentioned above (23), HUVECs, to put it simply, were obtained and spread on the 24-hole plate coated with Matrigel (BD Biosciences). Photos of the cells were taken with an infinity-corrected optical element under a Nikon Eclipse microscope. The WimTube software (WIMASIS, Munich, Germany, 2015, Image Analysis) was harnessed to analyze and explain the tubule formation assay in light of different parameters.

### Flow Cytometry (FCM)

Phorbol-12-Myristate-13-acetate (PMA, 100 ng/mL; Catalog No. P1585; Sigma-Aldrich; Merck KGAA) was taken to differentiate THP-1 cells (24). The differentiated THP-1 cells were dyed with FITC-CD14, PE-CD11b, PE-F4/80, and FITC-CD11c antibodies (BD Biosciences) and incubated in darkness at indoor temperature for 15 minutes. Eventually, flow cytometry (BD Biosciences) analyzed the stained cells with the help of FACS Calibur Flow Cytometer (BD Biosciences, Sanjose, CA, USA). The whole process was conducted in triplicate.

### Tumor Xenotransplantation Model

In order to detect the influence of CALM2 *in vivo*, the lentivirus vectors of CALM2, si-CALM2, and their controls were stably transfected into luciferase-labeled AGS cells (1×10^6^), and the transfected vectors were subcutaneously transfused into the left and right sides of the female BALB/c nude mice (n=5, 3-4 weeks old, bought from the Animal Center of the Chinese Academy of Sciences) for tumor growth observation. The volume of the tumors was measured on the 7^th^, 12^th^, 17^th^, 22^nd^, 27^th^, and 32^nd^ days after the injection. On the 35^th^ day, the mice were dislocated and killed, with their tumors excised and the tumor volume (mm^3^) and mass (g) measured. Tumor volume was calculated in line with this formula: volume = length × width^2^ × 0.5. AGS cells, stably transfected with the lentivirus vectors of CALM2, Si-CALM2, and their controls, were flushed in PBS and resuspended at 1×10^7^ cells/mL. The AGS cell suspension (0.1 mL) was injected into the mice’s tail veins. The animals were killed seven weeks after the transfusion. The lung tissues were taken out and photographed, and the visible tumors on the surface were tallied. IHC analysis was done on the collected lung tissues. All of the animal procedures were granted by the ethics committee of People’s Hospital of Wuhan University. We tried our best to limit the suffering of mice.

### Statistical Analysis

The SPSS17.0 statistical software (SPSS Inc., Chicago, IL, USA) was deployed for analysis. Measurement statistics were displayed as mean ± standard deviation (x ± s). Person analysis was taken to analyze CALM2’s affinities with CD163, CD206, and CD11b. Kaplan-Meier curve examined the relationship of CALM2 expression with the cumulative survival time of tumor patients. ANOVA was adopted to compare multiple groups, and an independent sample t-test was introduced to compare two groups. P<0.05 was deemed to be statistically significant.

## Results

### CALM 2 Up-Regulation Was Correlated With Poorer GC Prognosis

**Figure 1:**
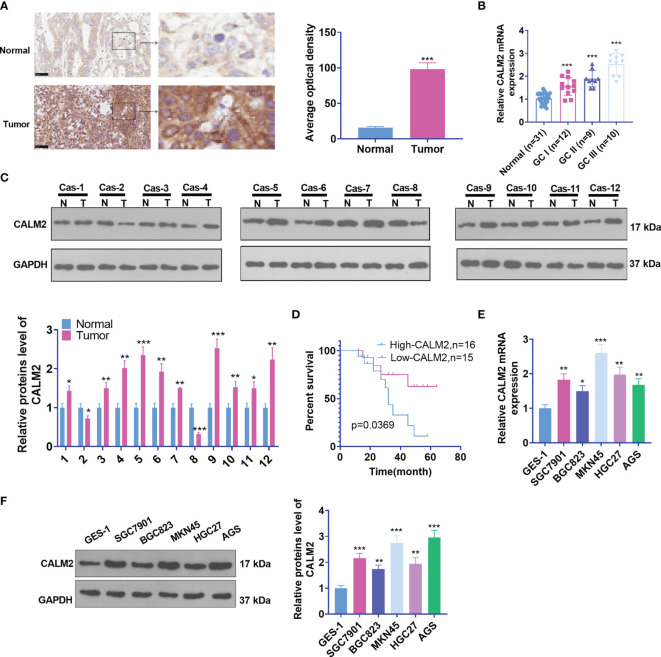
CALM2 up-regulation was correlated with poorer GC prognosis. **(A)** CALM2 expression in GC and paired para-carcinoma tissues was figured out through IHC staining. **(B)** qRT-PCR determined the mRNA level of CALM2 in GC tissues of patients in different clinical phases. **(C)** The protein level of CALM2 in GC tissues of 12 different GC tissues was evaluated through Western Blot. **(D)** Kaplan-Meier curve analyzed the correlation between the high or low profile of CALM2 and the survival rate of 31 patients with GC. p = 0.0315. **(E)** CALM2 mRNA expression in the human gastric epithelial cell line GES-1 and gastric cancer cell lines SGC7901, BGC823, MKN45, HGC27, as well as AGS was confirmed *via* qRT-PCR. **(F)** Western Blot measured the protein profile of CALM2 in different GC cell lines. Statistics were displayed as mean ± SD (n = 3). *P < 0.05, **P < 0.01, ***P < 0.001.

**Figure 2:**
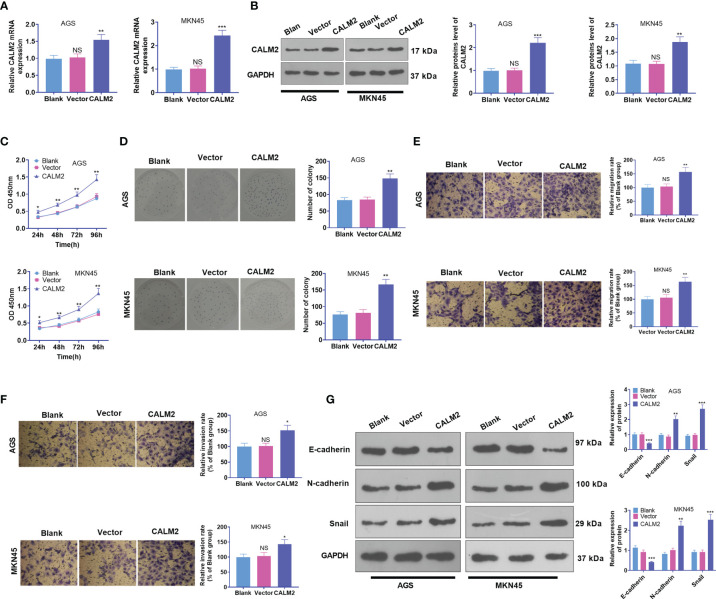
CALM2 overexpression strengthened GC cell proliferation, migration, and invasion. CALM2 overexpression plasmids or negative vectors were transfected into AGS and MKN45 cells. **(A)** qRT-PCR ascertained the mRNA expression of CALM2 in AGS and MKN45 cells. **(B)** Western Blot determined the protein level of CALM2 in AGS and MKN45 cells. **(C)** The proliferation of AGS and MKN45 cells was monitored by CCK8. **(D)** The colony formation capability of AGS and MKN45 cells was verified *via* colony formation experiment. **(E, F)** Transwell evaluated the migration and invasion of AGS and MKN45 cells. **(G)** Western Blot was adopted to examine the EMT-concerned proteins of the cells. Data were presented as mean ± SD (n = 3). *P < 0.05, **P < 0.01, ***P < 0.001 (*vs.* the Vector group). NSP > 0.05 (vs. Blank group).

To understand the biological function of CALM2, we first examined CALM2 expression in GC tissues and para-carcinoma tissues of 31 IHC cases. As shown by the results, CALM2 expression was uplifted in GC tissues (**Figure 1A**). qRT-PCR ascertained its expression in GC tissues of patients in different clinical phases. Its profile was relevant to clinical GC stages. CALM2 presented the highest expression during the GC III stage (**Figure 1B**). Western Blot measured CALM2 expression in 12 GC tissues, indicating that by contrast to para-carcinoma tissues, CALM2 was highly expressed in GC tissues (**Figure 1C**). The overall survival rate of patients with high CALM2 expression was remarkably lower than that of patients with low CALM2 expression (**Figure 1D**). Then, qRT-PCR and Western Blot (WB) both confirmed CALM2’s expression in various GC cell lines, signifying that in contrast with the gastric epithelial cell line GES-1, CALM2 had a high profile in GC cell lines (**Figures 1E, F**). Moreover, we verified CALM2 level in stomach adenocarcinoma (STAD) *via* the GEPIA database (http://gepia.cancer-pku.cn/). It was disclosed that CALM2 was vigorously up-regulated in STAD tissues vis-a-vis normal stomach tissues (**Figure 1A**). CALM2 up-regulation in STAD was also confirmed by IHC data from the Human Protein Atlas (https://www.proteinatlas.org/) (**Figures 1B, C**). The prognostic value of CALM2 in STAD patients was determined through Kaplan-Meier Plotter (http://kmplot.com/analysis/), which signified that higher CALM2 level foreboded poorer overall survival and first progression survival of STAD patients instead of post progression survival (**Figures 2A–C**). Given these outcomes, the profile of CALM2 was augmented in GC tissues and cells, which was associated with poor GC prognosis.

### CALM2 Overexpression Stepped Up GC Cells’ Proliferation, Migration, and Invasion

CALM2 expression in GC cells came to light, but the specific role of CALM2 in the cells remained obscure. Thus, Vector and CALM2 overexpression plasmid were transfected into AGS and MKN45 cells. qRT-PCR and Western Blot revealed that CALM2 exhibited a high profile in GC cells, which verified the success in the transient transfection (**Figures 2A, B**). Next, GC cell proliferation was confirmed through CCK8 and colony formation assay. By contrast to the Vector group, the proliferation and colony formation ability of GC cells in the CALM2 group were strengthened (**Figures 2C, D**). Transwell pinpointed that in comparison with the Vector group, GC cells’ migration and invasion in the CALM2 group were bolstered (**Figures 2E, F**). Western Blot checked the profiles of EMT-concerned proteins E-cadherin, N-cadherin, and Snail in GC cells, uncovering that in contrast with the Vector group, E-cadherin was down-regulated while N-cadherin and Snail were up-regulated in the CALM2 group (**Figure 2G**). All the findings denoted that CALM2 exerted a cancer-promoting function in GC.

### CALM2 Down-Regulation Hampered GC Cell Proliferation, Migration, and Invasion

**Figure 3:**
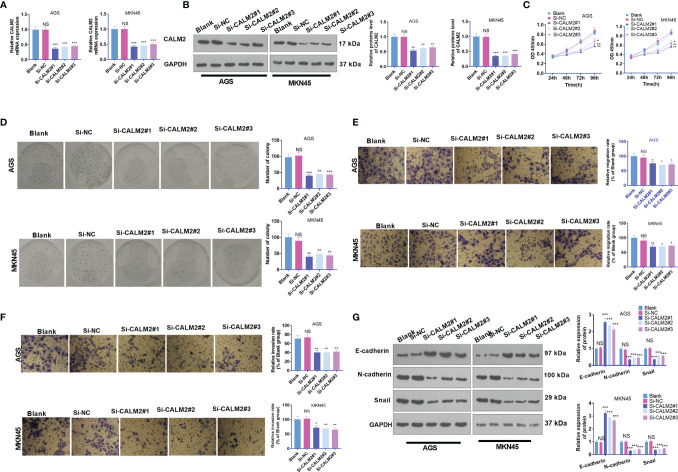
CALM2 inhibition hampered GC cell proliferation, migration, and invasion. Si-NC, si-CALM2#1, si-CALM2#2, or si-CALM2#3 was transfected into AGS and MKN45 cells. **(A)** qRT-PCR checked the mRNA expression of CALM2 in AGS and MKN45 cells. **(B)** The protein level of CALM2 in AGS and MKN45 cells was examined through Western Blot. **(C)** The proliferation of AGS and MKN45 cells was monitored through CCK8. **(D)** Colony formation assay determined the colony formation ability of AGS and MKN45 cells. **(E, F)** Transwell measured these cells’ migration and invasion. **(G)** EMT-associated proteins in AGS and MKN45 cells were examined *via* Western Blot. Statistics were displayed as mean ± SD (n=3). *P < 0.05, **P < 0.01, ***P < 0.001 (*vs.* the Si-NC group). NS *P* > 0.05 (vs. Blank group).

To further demonstrate the function of CALM2 in GC cells, we transfected si-NC, si-CALM2#1, si-CALM2#2, and si-CALM2#3 into GC cells. It was unraveled that the mRNA and protein levels of CALM2 in GC cells transfected with si-CALM2#1, si-CALM2#2, and si-CALM2#3 were lowered against the Si-NC group (**Figures 3A, B**). In view of CCK8 assay, colony formation assay, and Transwell assay, CALM2 knockdown repressed GC cell proliferation (**Figure 3C**), colony formation (**Figure 3D**), migration (**Figure 3E**), and invasion (**Figure 3F**). CALM2 knockdown also impeded EMT-concerned processes in the cells (**Figure 3G**). As a result, the above data reflected that CALM2 down-regulation exerted anti-tumor effects on GC.

### CALM2 Overexpression Strengthened GC Cell-Mediated Angiogenesis and “M2” Macrophage Polarization

**Figure 4:**
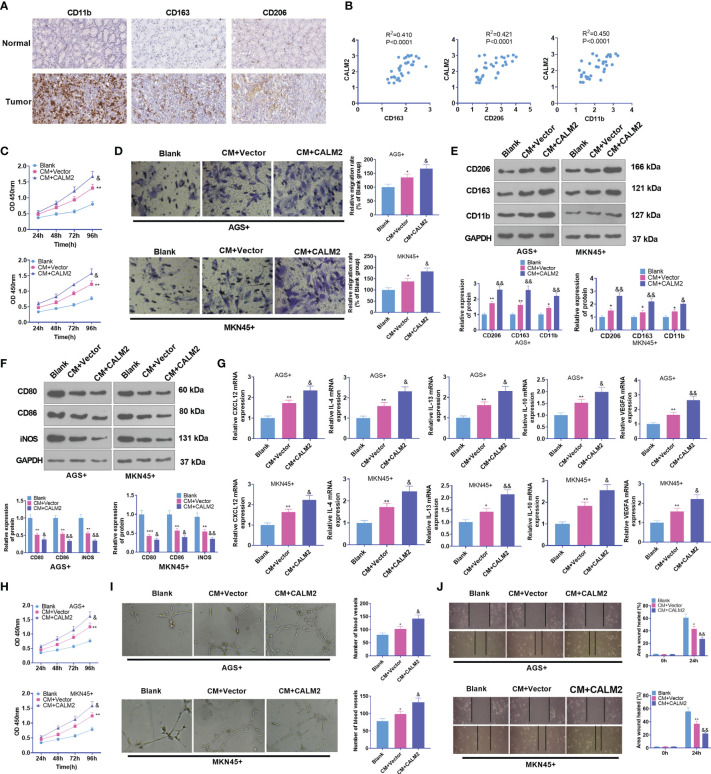
CALM2 overexpression aggravated GC cell-mediated angiogenesis and macrophage polarization. **(A)** IHC was operated to assess the profiles of CD163, CD206, and CD11b in GC tissues (data from the Human Protein Atlas). **(B)** CALM2’s affinities with CD163, CD206, and CD11b in GC tissues were analyzed through Person regression analysis. THP1 cells stimulated by PMA were co-cultured with the conditioned medium of GC cells. **(C)** CCK8 monitored macrophage proliferation. **(D)** Transwell investigated macrophage migration. **(E, F)** The profiles of M2-type macrophage surface receptors CD206, CD163, and CD11b and M1-type macrophage markers CD80, CD86, and iNOS were figured out *via* Western Blot. **(G)** The mRNA expressions of M2-type macrophage-concerned factors CXCL12, IL-4, IL-13, IL-10, and VEGFA were confirmed through qRT-PCR. HUVECs were co-cultured with the conditioned medium of GC cells. **(H)** The viability of HUVECs was examined by CCK8. **(I)** HUVEC angiogenesis was checked by tubule formation experiment. **(J)** HUVECs’ migration was assessed through the wound scratch test. Statistics were presented as mean ± SD (n=3). *P < 0.05, **P < 0.01, ***P < 0.001 (vs. the Blank group). *&P < 0.05, &&P < 0.01* (*vs.* the CM+Vector group).

Macrophages are the most frequently seen cells in the tumor stroma. Tumor-associated macrophages usually refer to a class of M2-type macrophages equipped with pro-tumor effects (25). Therefore, M2-type macrophage markers in GC tissues, covering CD163, CD206, and CD11b, were analyzed through IHC statistics obtained from the human protein atlas (https://www.proteinatlas.org/). It was uncovered that in contrast with para-carcinoma tissues, immune responses of CD163, CD206, and CD11b were much stronger in GC tissues (**Figure 4A**). Through Person analysis, we discovered that CALM2 was positively correlated with CD163, CD206, and CD11b (**Figure 4B**). To confirm the influence of CALM2 on macrophages at the cellular level, we administered 100 ng/mL PMA to spur macrophage differentiation in THP-1 monocytes. GC cells with Vector or CALM2 overexpression were cultivated for 24 hours to acquire the conditioned medium which was later cultured together with macrophages for 24 hours. As indicated by CCK8 and colony formation experiments, in comparison with the Blank group, the cell viability of macrophages was enhanced in the CM+Vector group and further stepped up by CALM2 overexpression (**Figure 4C**). Transwell signified that in contrast with the Blank group, macrophage migration was bolstered in the CM+Vector group. In comparison with the CM+Vector group, it was boosted by CALM2 overexpression (**Figure 4D**). Western Blot and qRT-PCR were taken to detect M1 macrophage markers CD86, CD80, and iNOS, M2 macrophage surface receptors CD206, CD163, and CD11b, and M2-correlated genes *CXCL12, IL-4, IL-13, IL-10*, and *VEGFA*. By contrast to the Blank group, the profiles of CD206, CD163, CD11b, *CXCL12, IL-4, IL-13, IL-10*, and *VEGFA* were raised, whereas the expressions of M1 markers CD80, CD86, and iNOS were attenuated in the CM-Vector group. In comparison with the CM+Vector group, M2 polarization markers were further enhanced, while M1 polarization markers were repressed by CALM2 overexpression (**Figures 4E–G**). To detail the pro-angiogenesis effect of GC cells on HUVECs, we cultivated the conditioned medium from GC cells with HUVECs for 24 hours. HUVEC proliferation and angiogenesis were examined through CCK8 and tubule formation experiment. It turned out that compared to the Blank group, the viability and angiogenesis of HUVECs in the CM+Vector group were boosted. In contrast with the CM+Vector group, they were further enhanced by CALM2 overexpression (**Figures 4H, I**). IHC outcomes, aligned with those of tubule formation assay, denoted that in contrast with the Blank group, angiogenic markers CD31 and VEGFA in HUVECs were markedly up-regulated in the CM+Vector group. In contrast with the CM+Vector group, the profiles of CD31 and VEGFA in HUVECs were augmented by CALM2 overexpression (**Figure 4J**). As per these discoveries, CALM2 overexpression stepped up macrophage polarization and angiogenesis, which were mediated by GC cells.

### CALM2 Facilitated GC Cell Growth and Metastasis *In Vivo*

**Figure 5:**
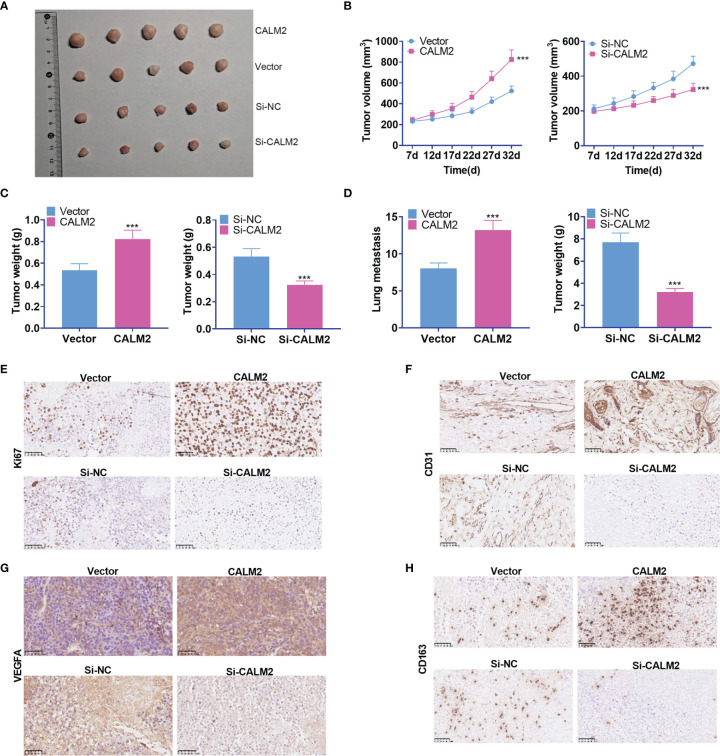
CALM2 overexpression or knockdown could facilitate or hinder tumor growth and metastasis *in vivo*. The nude mice were transplanted with AGS cells transfected with CALM2 overexpression plasmid or si-CALM2. After 35 days of neoplasm transplantation, the animals were sacrificed, with their tumors isolated. **(A)** The picture of the xenograft tumor. **(B)** Quantitative analysis of tumor size. **(C)** Quantitative analysis of tumor weight. **(D)** Quantitative analysis of the amount of lung metastatic nodules. **(E–H)** IHC analyzed and evaluated the profiles of Ki67, CD31, VEGFA, CD163 in the tumors. Statistics were displayed as mean ± SD (n = 5). ***P < 0.001 (*vs.* the Vector group or the Si-NC group).

To validate the function of CALM2 *in vivo*, we transfected the lentiviral vectors of CALM2 or si-CALM2 respectively into AGS cells stably. Transfected cells were subcutaneously transfused into the right and upper side of each mouse to set up a xenograft tumor model. It was disclosed that CALM2 overexpression considerably heightened the volume and weight of the tumors, which were nonetheless reduced by CALM2 knockdown (**Figures 5A–C**). To further explore the influence of CALM2 on lung metastasis *in vivo*, we injected AGS and MKN45 cells with CALM2 overexpression or knockdown into the tail veins of the mice. It transpired that CALM2 overexpression increased metastatic pulmonary nodules in the animals, whereas CALM2 knockdown remarkably abated them (**Figure 5D**). The xenograft tumors were measured through IHC. It was unraveled that CALM2 overexpression notably uplifted the profiles of the cell proliferation marker Ki67, angiogenesis-concerned genes CD31 and VEGFA, as well as the M2-type macrophage surface receptor CD163. On the other hand, CALM2 knockdown evidently lowered those of Ki67, CD31, VEGFA, CD163 (**Figures 5E–H**). It came to light that CALM2 played a carcinogenic role in tumor growth and metastasis *in vivo*.

### CALM2 Overexpression Activated the Profile of JAK2/STAT3/HIF-1/VEGFA in GC Tissues and Cells

**Figure 6:**
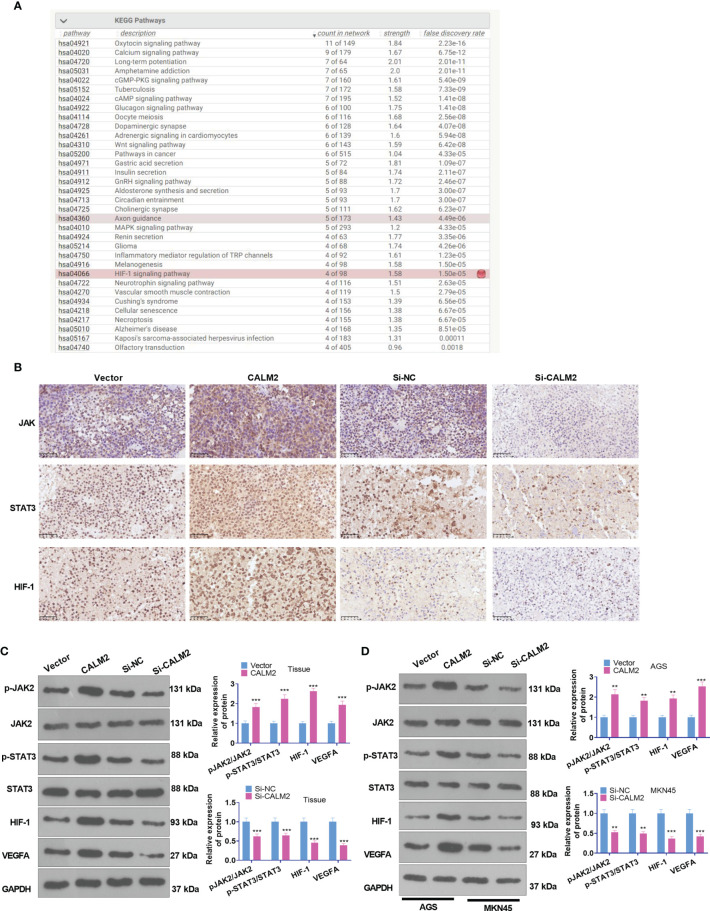
CALM2 overexpression activated JAK2/STAT3/HIF-1/VEGFA profile in GC tissues and cells. **(A)** String (https://string-db.org/) was taken for analyzing the potential pathway regulated by CALM2. **(B)**. JAK2/STAT3/HIF-1/VEGFA was discovered to be an underlying downstream pathway of CALM2. **(C)** IHC ascertained the protein profiles of p-JAK2, p-STAT3, and HIF-1A in the mice’s tumor tissues. **(C)** Western Blot confirmed the protein profile of the JAK2/STAT3/HIF-1/VEGFA signaling axis in AGS and MKN45 cells with CALM2 overexpression or down-regulation. Data were exhibited as mean ± SD (n=3). **P < 0.01, ***P < 0.001 (*vs.* the Vector or Si-NC group).

The function of CALM2 *in vivo* was fully understood, but its exact mechanism was still beyond our knowledge. Consulting String (https://string-db.org/), we discovered that Calcium signaling and HIF signaling and pathways in cancer were the underlying pathways of CALM2 (**Figure 6A**). The PPI network reflected that CALM2 potentially modulated the JAK2/STAT3/HIF-1/VEGFA axis *via* CAMK2A (PPI enrichment p-value=2.76e-06, **Figure 6B**). The relevant genes of CALM2 in STAD were examined *via* LinkedOmics (http://linkedomics.org/login.php). The enrichment analysis assessed the potential KEGG pathways of CALM2 in STAD, indicating that the Gastric cancer pathway is a promising pathway of CALM2 (**Supplementary Figures 3A–C**). And the KEGG pathway (http://www.kegg.jp/) signified that CALM2 might have a correlation with the JAK-STAT pathway (**Supplementary Figure 3D**). IHC and Western Blot determined the protein profile of JAK2/STAT3/HIF-1/VEGFA in the Xenograft tumors on mice and GC cells. It was revealed that in GC tumor tissues and GC cells, in contrast with the Vector group, CALM2 overexpression greatly facilitated JAK2 and STAT3 phosphorylation and elevated the protein expressions of HIF-1 and VEGFA. Nevertheless, CALM2 contributed to the opposite situation (**Figures 6C, D**). Consequently, CALM2 potentially modulated GC progression *via* the JAK2/STAT3/HIF-1A/VEGFA axis.

### JAK2 or HIF-1 Inhibition Weakened CALM2-Mediated Influence on GC Cell Proliferation, Migration, and Invasion

**Figure 7:**
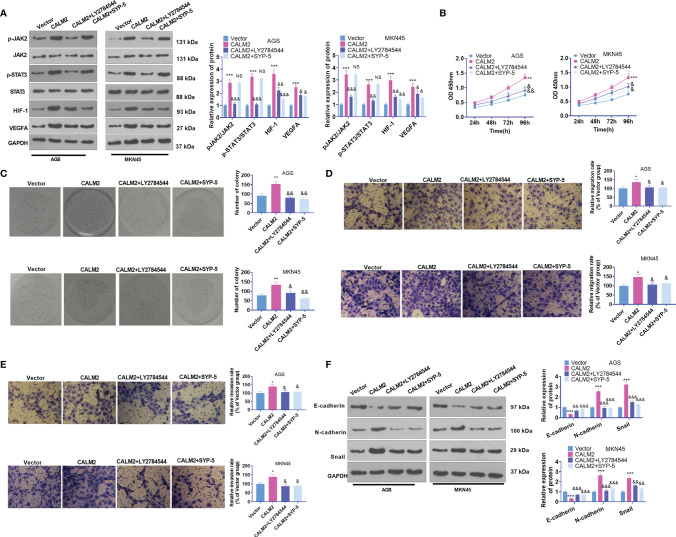
JAK2 or HIF-1 inhibition reduced CALM2’s impact on GC cell proliferation, migration, and invasion. The JAK2 inhibitor LY2784544 (1 μM) and HIF-1 inhibitor SYP-5 (10 μM) were applied to AGS and MKN45 cells with CALM2 overexpression for treatment. **(A)** Western Blot determined the profile of the JAK2/STAT3/HIF-1/VEGFA signaling axis in AGS and MKN45 cells. **(B)** The proliferation of those cells was monitored by CCK8. **(C)** Colony formation assay examined the colony formation of AGS and MKN45 cells. **(D, E)** Transwell measured the cells’ migration and invasion. **(F)** EMT-correlated proteins in AGS and MKN45 cells were examined through Western Blot. Statistics were displayed as mean ± SD (n=3). *P < 0.05, **P < 0.01, ***P < 0.001 (vs. the Vector group). *NSP > 0.05, &P < 0.05, &&P < 0.01, &&&P < 0.001* (*vs.* the CALM2 group).

To dig deeper into the role of the JAK2/STAT3/HIF-1/VEGFA signaling axis in GC, we utilized the JAK2 inhibitor LY2784544 (1 μM) and HIF-1 inhibitor SYP-5 (10 μM) in AGS and MKN45 cells with CALM2 overexpression. Western Blot figured out the profile of JAK2/STAT3/HIF-1/VEGFA. By contrast to the CALM2 group, the application of LY2784544 or SYP-5 substantially attenuated JAK2 and STAT3 phosphorylation and brought down HIF-1 and VEGFA expressions (**Figure 7A**). In contrast with the CALM2 group, the JAK2 or HIF-1 inhibitor considerably alleviated the effects of CALM2 on GC cells in the processes covering proliferation (**Figures 7B, C**), migration (**Figure 7D**), invasion (**Figure 7E**), and GC cell EMT progression (**Figure 7F**). Therefore, JAK2 or HIF-1A inhibition restrained the CALM2-mediated oncogenic function in GC cells.

### JAK2 or HIF-1 Inhibition Attenuated CALM2-Mediated Macrophage Polarization and Angiogenesis

**Figure 8:**
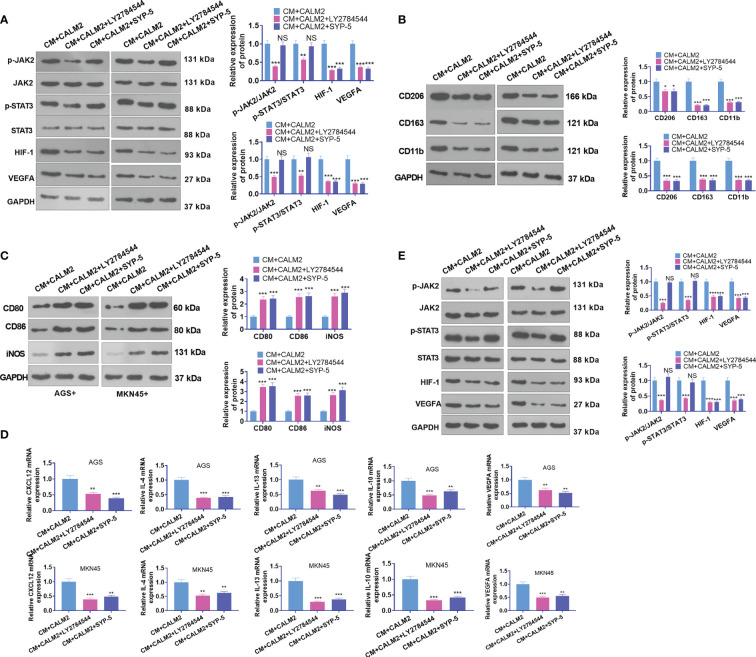
JAK2 or HIF-1 inhibition mitigated CALM2-mediated macrophage polarization and angiogenesis. The JAK2 inhibitor LY2784544 (1 μM) and HIF-1 inhibitor SYP-5 (10 μM) were deployed to treat AGS and MKN45 cells with CALM2 overexpression. The conditioned medium was collected and cultivated along with macrophages and HUVECs for 24 hours. **(A)** Western Blot was done to figure out the profile of the JAK2/STAT3/HIF-1/VEGFA axis in macrophages. **(B, C)** Western Blot also ascertained the protein profiles of CD206, CD163, CD11b, CD80, CD86, and iNOS in macrophages. **(D)** qRT-PCR disclosed the mRNA expressions of M2-type macrophage-concerned factors CXCL12, IL-4, IL-13, IL-10, and VEGFA. **(E)** Western Blot verified the profile of the JAK2/STAT3/HIF-1/VEGFA pathway in HUVECs. Data were presented as mean ± SD (n=3). NSP > 0.05, *P < 0.05, **P < 0.01, ***P < 0.001 (*vs.* the CM+CALM2 group).

AGS and MKN45 cells transfected with CALM2 overexpression plasmid were treated with the JAK2 inhibitor LY2784544 (1 μM) and HIF-1 inhibitor SYP-5(10 μM) for 24 hours. The conditioned medium was collected and cultivated along with macrophages for 24 hours. The profile of JAK2/STAT3/HIF-1/VEGFA in macrophages was figured out through Western Blot. It was discovered that in comparison with the CM+ CALM2 group, the use of LY2784544 and SYP-5 weakened JAK2 and STAT3 phosphorylation in macrophages, whereas the profiles of HIF-1 and VEGFA were brought down (**Figure 8A**). WB reflected that in contrast with the CM+CALM2 group, the protein profiles of CD206, CD163, and CD11b in macrophages were lowered after LY2784544 and SYP-5 treatment. Notwithstanding, LY2784544 and SYP-5 treatment enhanced the profiles of CD80, CD86, and iNOS (**Figures 8B, C**). qRT-PCR signified that the mRNA expressions of CXCL12, IL-4, IL-13, IL-10, and VEGFA in macrophages declined following the use of LY2784544 and SYP-5, vis-a-vis the CM+CALM2 group (**Figure 8D**). Similarly, to examine the function of the JAK2/STAT3/HIF-1/VEGFA axis in HUVECs, we collected the conditioned medium of GC cells and cultured it with HUVECs for 24 hours. Through Western Blot, it was uncovered that in contrast with the CM+CALM2 group, JAK2 and STAT3 phosphorylation in macrophages were weakened, and the profiles of HIF-1 and VEGFA were brought down subsequent to the administration of LY2784544 and SYP-5 (**Figure 8E**). In comparison with the CM+CALM2 group, LY2784544 and SYP-5 treatment substantially reduced HUVEC proliferation and angiogenesis (**Figure 8E**). The above outcomes indicated that JAK2 or HIF-1 inhibition attenuated the CALM2-incurred boosting effects on macrophage polarization and angiogenesis mediated by GC cells.

## Discussion

Gastric cancer is regarded as the second most common cause of cancer-concerned death in the world. Given the poor prognosis of patients suffering from advanced gastric cancer, new strategies must be developed to raise the survival rate of the disease (26). Therefore, a probe into the molecular mechanism in GC is essential. Here, CALM2 was discovered to display a high expression in GC tissues and cells, which correlated with poor prognosis and malignant tumor progression. In *in-vivo* trials, it was also disclosed that highly-expressed CALM2 could facilitate tumor growth. These findings hinted that CALM2 might be an oncogene in GC, boosting the malignant development of GC cells.

TME is a sophisticated system accommodating multiple cells and cytokines. Among a variety of immune cells, the exceptionally abundant macrophages exert an important function in the whole process of tumor development (27). TAM usually refers to M2-type macrophages boasting anti-inflammatory and pro-tumor functions (25). It has been uncovered that TAM separated from GC tissues mainly displays the M2 phenotype, and enhanced M2 macrophage polarization can notably facilitate malignant GC progression (28–30). The study demonstrated that the profiles of M2-phenotype markers CD163, CD206, and CD11b in the tissues were apparently elevated. That is to say, M2-type macrophage polarization may be critical to GC progression.

It has been revealed that CALM2 mutation bears a relation to susceptibility to congenital arrhythmia, which has been extensively investigated in LONG QT syndrome (31). As displayed by recent studies, CALM2 exhibits an aberrant expression in breast cancer cells (32) and neuroblastoma (33), but its certain function in cancer remains a mystery. More significantly, in GC cells, Cai H et al. have uncovered that CALM2 is a target of hsa-miR-19b/hsa-miR-181b concerning GC, maintaining that CALM2 may be an indispensable prognostic molecule for GC (34). CALM2 may bolster the activation of calcium/calmodulin-dependent kinase2 (CAMK2) and up-regulate MMP-9 production mediated by NF-κB and Akt, hence stepping up gastric cancer metastasis (35). CALM2 boosts CAMK2 and arouses the AMPK signaling axis, therefore increasing the proliferation, colony formation, and invasion of gastric cancer cells (36). It can be concluded that CALM2 can serve as a cancer promoter in GC. Nonetheless, we are still in the dark about the influence of CALM2 on macrophages in GC. Here, we figured out that CALM2 presented a high expression in GC tissues and cells, and such a high expression augmented GC cell proliferation, migration, and invasion. The discovery is in line with prior works. Furthermore, it was revealed that highly-expressed CALM2 boosted macrophage polarization, enhanced macrophage proliferation, migration, and invasion, and facilitated endothelial cell angiogenesis. The findings demonstrated the cancer-promoting function of CALM2 in GC.

STAT3, a crucial member of STAT proteins, is one of the most prevailing oncogenes in human cancers, acting as a cancer booster in GC development (37). JAK2 is pivotal to cytokine receptor signaling, and JAK2 kinase phosphorylates STAT3. JAK2/STAT3 activation inhibition can hamper GC proliferation, migration, and invasion (16, 38). It is widely acknowledged that macrophage polarization pertains to tumor development (39). What’s more, STAT3 pathway activation induces macrophage M2 polarization, thus aggravating tumorigenesis. For instance, tumor-derived Leukemia inhibitory factor (LIF) transcription is enhanced by HIF1α signaling activation following cisplatin treatment. Tumor cell-derived LIF stimulates macrophages’ M2-type polarization through activating the STAT3 signaling pathway (40). JAK2/STAT3 inhibition can dampen the polarization of M2-type macrophages and slow down the progression of cancer. For instance, oleanolic acid can curb M2 polarization of macrophages and proliferation of tumor cells in glioblastomas *via* restraining STAT3 activation (41).

Angiogenesis is a symbol of cancer (42). VEGFA is a critical mediator of tumor angiogenesis and a well-characterized target of HIF-1. The research displayed that HIF-1 and VEGFA were highly expressed in tumors, and restraint on the profiles of HIF-1 and VEGFA could frustrate angiogenesis (43, 44). The HIF-1/VEGFA axis has been uncovered to modulate macrophage M2-type polarization. For instance, In colorectal cancer, hypoxia stimulates M2 macrophage infiltration (45). Hepatocellular carcinoma cells compete with macrophages for iron. A lower iron environment steps up the M2 polarization of mouse macrophages through augmenting HIF-1α expression (46). What’s more, VEGFA up-regulation is also correlated with macrophage “M2” polarization (47, 48). Nonetheless, we still have no idea about the functions of HIF-1 and VEGFA in GC. Here, it was demonstrated that CALM2 overexpression uplifted JAK2/STAT3/HIF-1/VEGFA expression in GC tissues and cells. JAK2 or HIF-1 inhibition abated CALM’s influence on GC cell proliferation, migration, and invasion and also weakened its promotion on macrophage polarization and angiogenesis mediated by GC. These findings reflected that CALM2 could modulate the JAK2/STAT3/HIF-1/VEGFA signaling axis, hence impeding malignant GC cell development.

To conclude, our work has verified that CALM2 displays a high expression in GC tissues and cells and plays a carcinogenic role in the disease. Mechanically speaking, GC progression can be boosted by CALM up-regulating the JAK2/STAT3/HIF-1/VEGFA axis. CALM2 inhibition may be an underlying molecular target for targeted GC therapy.

## Data Availability Statement

The datasets presented in this study can be found in online repositories. The names of the repository/repositories and accession number(s) can be found in the article/**Supplementary Material**.

## Ethics Statement

The studies involving human participants were reviewed and approved by the ethic committee of People’s Hospital of Wuhan University. The patients/participants provided their written informed consent to participate in this study. Written informed consent was obtained from the individual(s), and minor(s)’ legal guardian/next of kin, for the publication of any potentially identifiable images or data included in this article.

## Author Contributions

Conceived and designed the experiments: HL. Performed the experiments: GM and YZ. Statistical analysis: ZD, LS, and YD. Wrote the paper: GM. All authors contributed to the article and approved the submitted version.

## Funding

This study was supported by grants from the National Natural Science Foundation of China (No. 81602116) and Hubei Provincial Natural Science Foundation (No. 2016CFB195).

## Conflict of Interest

The authors declare that the research was conducted in the absence of any commercial or financial relationships that could be construed as a potential conflict of interest.

## Publisher’s Note

All claims expressed in this article are solely those of the authors and do not necessarily represent those of their affiliated organizations, or those of the publisher, the editors and the reviewers. Any product that may be evaluated in this article, or claim that may be made by its manufacturer, is not guaranteed or endorsed by the publisher.

## Supplementary Material

Supplementary Figure 1 CALM2 had a higher level in stomach adenocarcinoma (STAD) **(A)**. CALM2 level in STAD was analyzed *via* the GEPIA database (http://gepia.cancer-pku.cn/). **(B, C)** CALM2 up-regulation in STAD was confirmed by IHC data acquired from The Human Protein Atlas (https://www.proteinatlas.org/).

Supplementary Figure 2 CALM2 was of prognostic value in STAD patients. **(A–C)** The prognostic value of CALM2 in STAD patients was examined by Kaplan-Meier Plotter (http://kmplot.com/analysis/), which indicated that higher CALM2 level predicted poorer overall survival **(A)** and first progression survival **(B)** of STAD patients rather than post progression survival **(C)**.

Supplementary Figure 3 CALM2 was potentially involved in gastric cancer progression *via* the CALM and JAK-STAT pathways. The relevant genes of CALM2 in STAD were examined *via* LinkedOmics (http://linkedomics.org/login.php). **(A)** The volcano plots of CALM2-correlated genes. **(B)** The heat map of positively-associated genes and negatively-related genes of CALM2 in STAD. **(C)** The enrichment analysis confirmed the underlying KEGG pathways of CALM2 in STAD. **(D)** The KEGG pathway (http://www.kegg.jp/) denoted that CALM2 manifested potential affinities with the JAK-STAT pathway.
