# Supplementary figures and images for: Calmodulin 2 Facilitates Angiogenesis and Metastasis of Gastric Cancer via STAT3/HIF-1A/VEGF-A Mediated Macrophage Polarization
Source: Front Oncol. 2021 Sep 15;11:727306. doi: 10.3389/fonc.2021.727306 (PMC8479158; doi:10.3389/fonc.2021.727306)

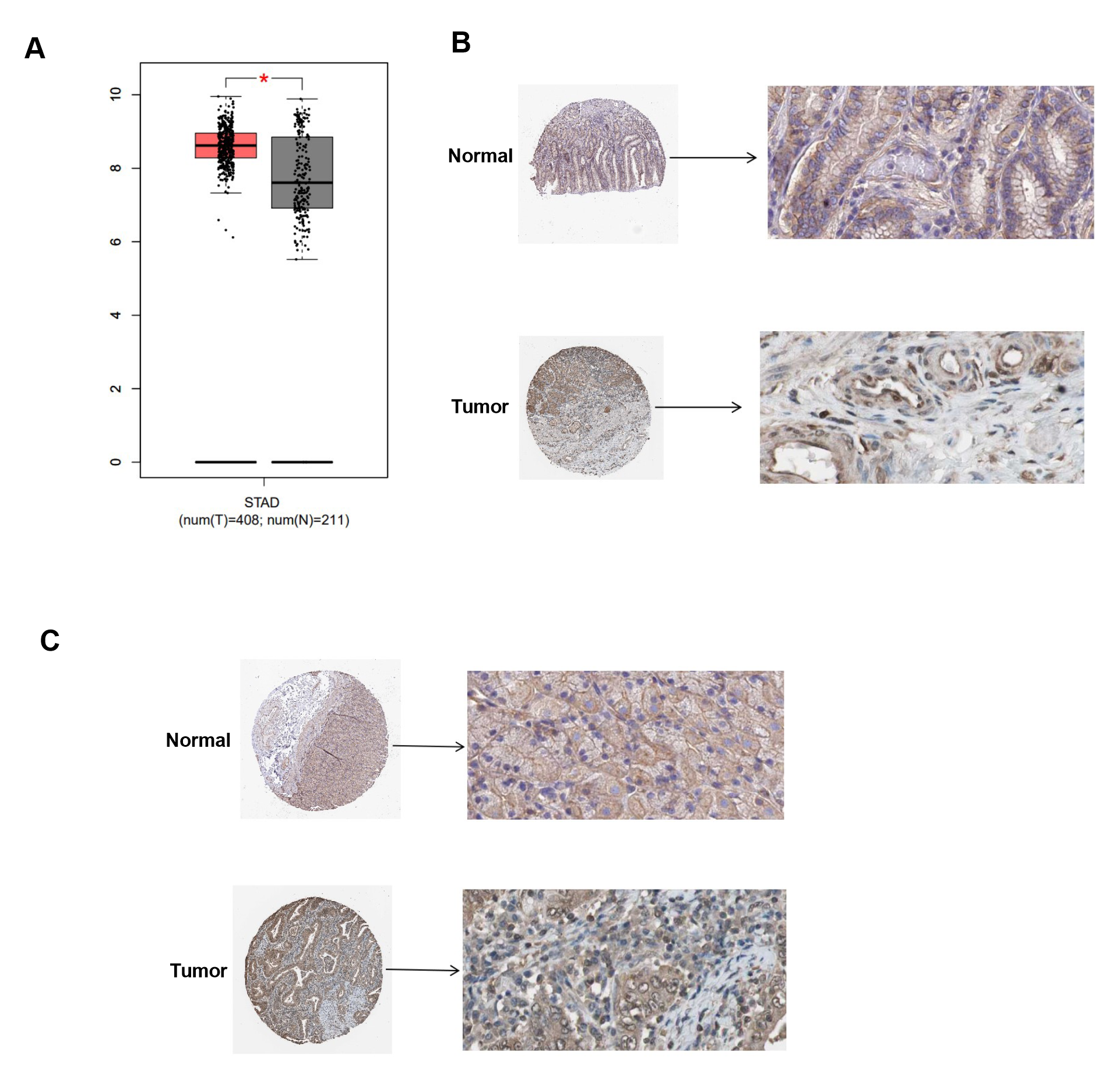

Supplement: Supplementary Figure 1 — CALM2 had a higher level in stomach adenocarcinoma (STAD) (A). CALM2 level in STAD was analyzed via the GEPIA database (http://gepia.cancer-pku.cn/). (B, C) CALM2 up-regulation in STAD was confirmed by IHC data acquired from The Human Protein Atlas (https://www.proteinatlas.org/). [file Image_1.tif]

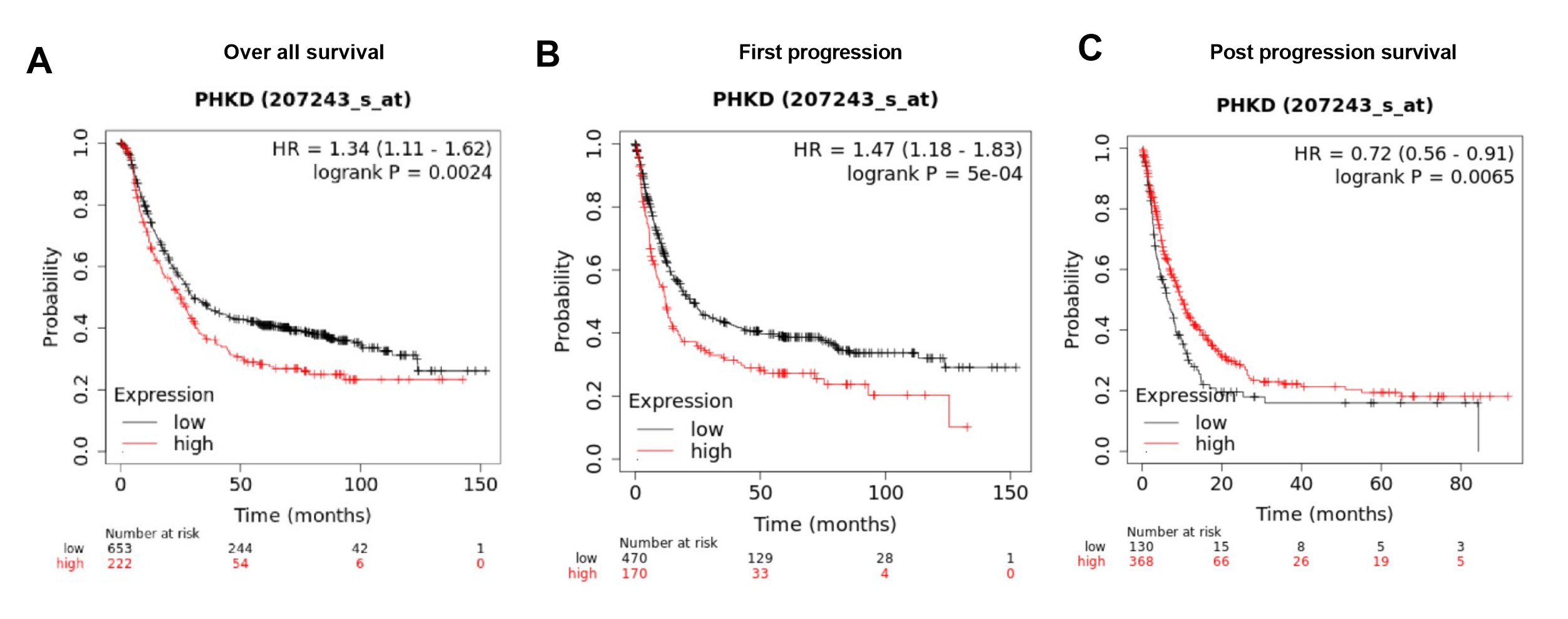

Supplement: Supplementary Figure 2 — CALM2 was of prognostic value in STAD patients. (A–C) The prognostic value of CALM2 in STAD patients was examined by Kaplan-Meier Plotter (http://kmplot.com/analysis/), which indicated that higher CALM2 level predicted poorer overall survival (A) and first progression survival (B) of STAD patients rather than post progression survival (C). [file Image_2.tif]

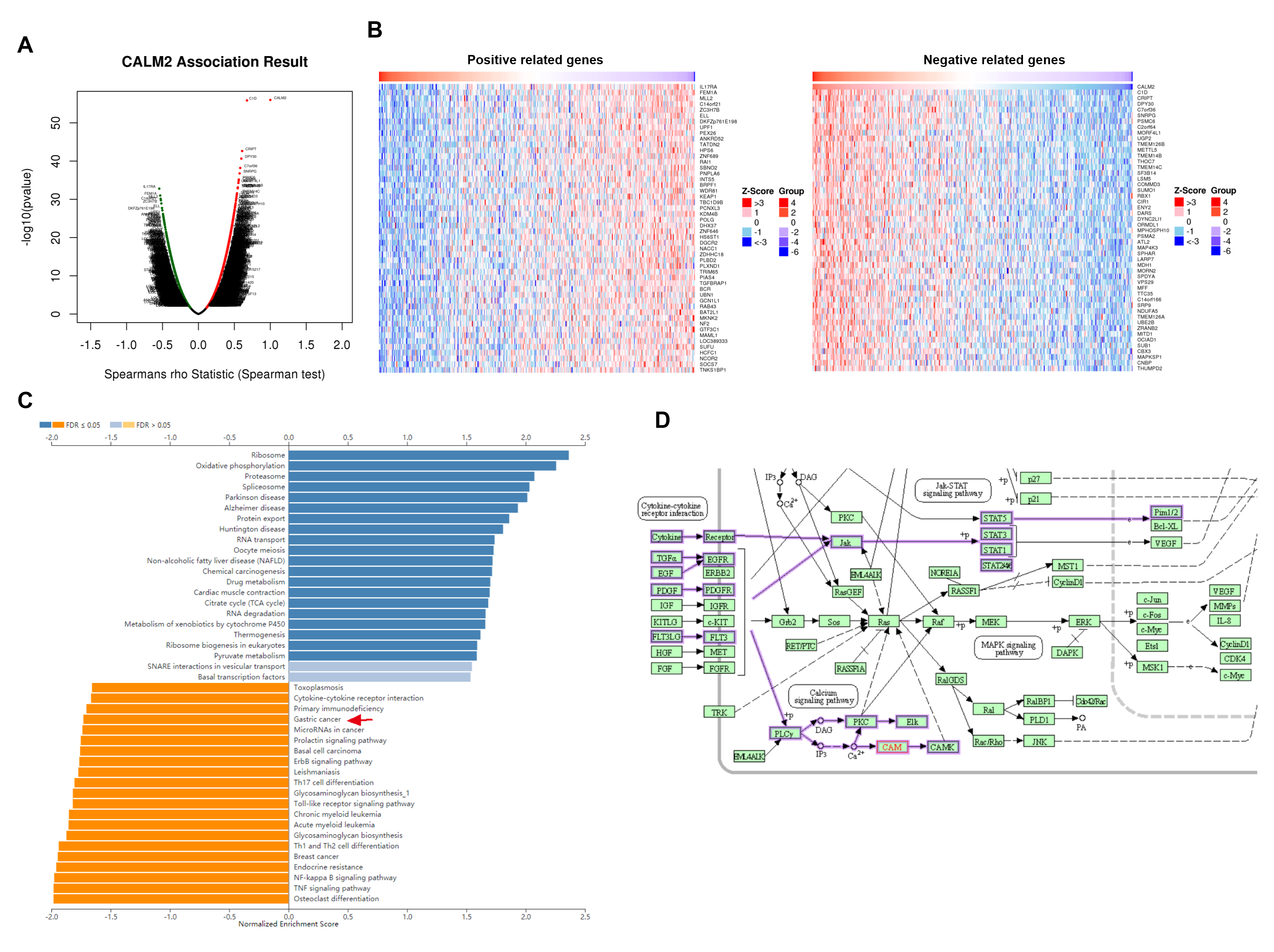

Supplement: Supplementary Figure 3 — CALM2 was potentially involved in gastric cancer progression via the CALM and JAK-STAT pathways. The relevant genes of CALM2 in STAD were examined via LinkedOmics (http://linkedomics.org/login.php). (A) The volcano plots of CALM2-correlated genes. (B) The heat map of positively-associated genes and negatively-related genes of CALM2 in STAD. (C) The enrichment analysis confirmed the underlying KEGG pathways of CALM2 in STAD. (D) The KEGG pathway (http://www.kegg.jp/) denoted that CALM2 manifested potential affinities with the JAK-STAT pathway. [file Image_3.tif]
